# Vesicle inhibition reduces *Candida* biofilm resistance

**DOI:** 10.1128/aac.00045-25

**Published:** 2025-03-26

**Authors:** Min-Ju Kim, Robert Zarnowski, Ryley Jones, Jeniel E. Nett, David Andes

**Affiliations:** 1 Department of Medicine, University of Wisconsin-Madison 200889, Madison, Wisconsin, USA; 2 Department of Medical Microbiology and Immunology, University of Wisconsin-Madison 5228, Madison, Wisconsin, USA; 3 William S Middleton VA Hospital 20132, Madison, Wisconsin, USA; University Children's Hospital Münster, Münster, Germany

**Keywords:** *Candida albicans*, biofilms, antifungal resistance, vesicles, extracellular matrix

## Abstract

*Candida* biofilm matrix components are delivered to the extracellular space by vesicles where they deposit and confer biofilm-associated drug resistance. Here, we present evidence that drugs designed to inhibit mammalian exosome production exhibit similar effects on *C. albicans* extracellular vesicles*,* ultimately eliminating biofilm matrix assembly. We find that vesicle reduction renders biofilm communities susceptible to the antifungal fluconazole. Our findings argue that vesicle trafficking pathways represent a promising target to optimize for recalcitrant fungal biofilms.

## INTRODUCTION

Microorganisms exist predominantly in surface-associated communities called biofilms (1). Microbes growing in this state are notable for their resistance to antimicrobials. *Candida albicans*, the most common healthcare-associated fungal infection, frequently forms biofilms on medical devices leading to disseminated disease and high mortality (2, 3). Treatment recalcitrance is so substantial that treatment guidelines recommend removal of *Candida*-infected devices (4). The intrinsic resistance of biofilms is multifactorial but largely attributable to a vesicle-delivered extracellular matrix that shields biofilm cells (5–7).

We recently identified an antifungal, turbinmicin, that inhibits fungal vesicular trafficking (8). We found that turbinmicin reduced *C. albicans* biofilm matrix delivery and rendered the biofilm subsequently susceptible to other antifungals (9). Numerous inhibitors of mammalian vesicle biogenesis have been identified and studied primarily in the context of cancer therapeutics. A recent review of this topic included dozens of such pharmacologic inhibitors, including several FDA-approved drugs commonly utilized for management of both cancer and non-cancer disease states (10). We reasoned that these agents may also disrupt vesicle delivery in related eukaryotic *Candida* biofilms, impairing matrix assembly and biofilm-associated drug resistance. We selected five drugs based upon commercial availability and inhibition of either exosome biogenesis (simvastatin, imipramine, glyburide, indomethacin) or release (omeprazole) (11–15).

**Fig 1:**
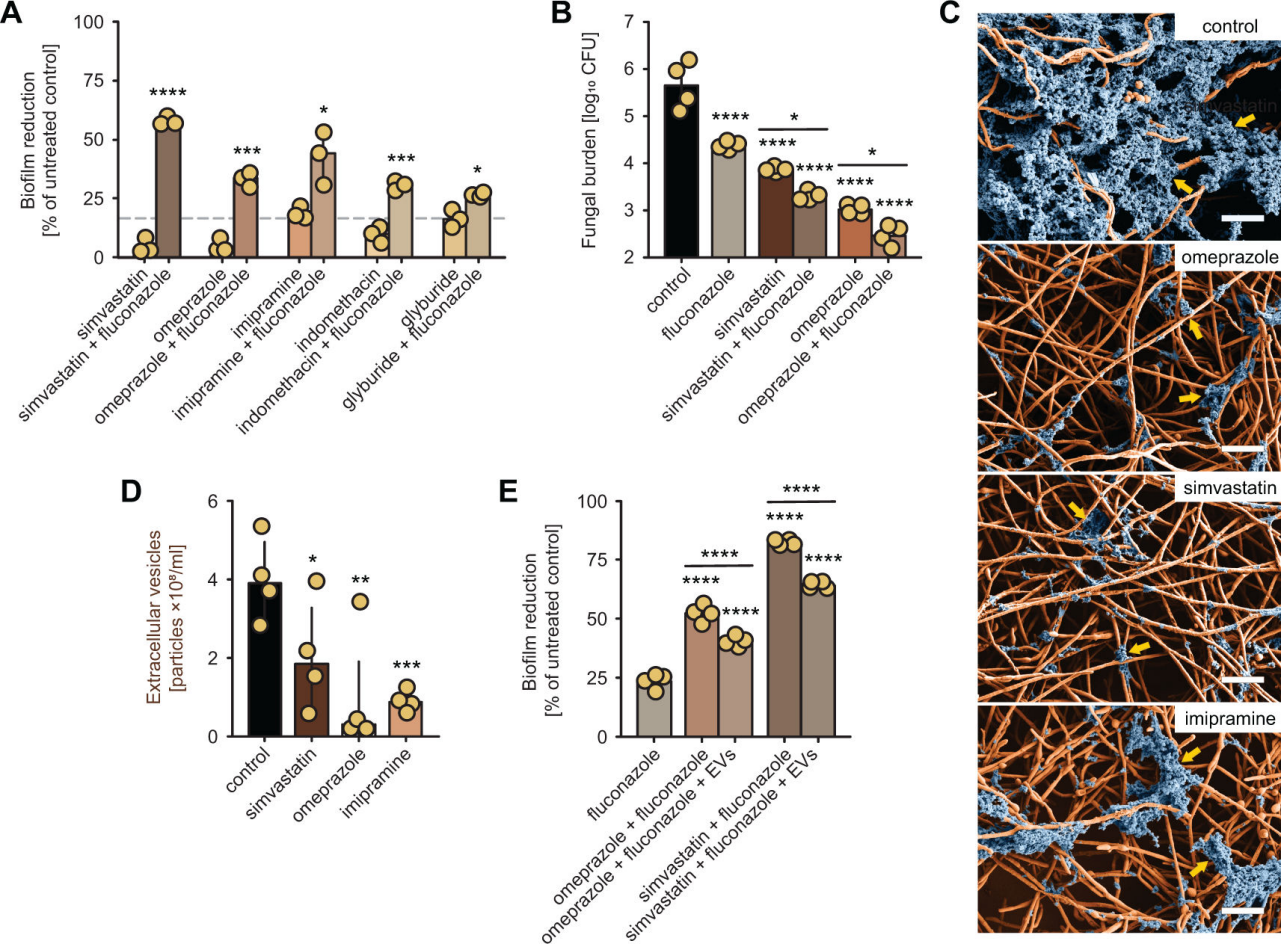
Impact of EV inhibitors on *C. albicans* biofilms. (A) The percent reduction in biofilm formation was measured using the XTT assay following treatment with either fluconazole (1,000  µg/mL) or a human EV inhibitor (8 µg/mL) [simvastatin, omeprazole, imipramine, indomethacin, or glyburide] alone or combined, compared to untreated biofilms. Each dot represents an independent biological replicate and reflects the mean of three technical replicates. Error bars denote standard deviation. A nonparametric Kruskal–Wallis one-way analysis of variance with an uncorrected Dunn’s multiple comparison test was performed. Indicated *P* values: *, *P* <0.05; **, *P* <0.01; ***, *P* <0.005; ****. (B) Quantification of *in vivo* biofilms using the rat central venous catheter model following antifungal therapy with either fluconazole (250  µg/mL) or a human EV inhibitor (32  µg/mL) [simvastatin or omeprazole] alone or in combination, compared to 0.9 M NaCl followed by the CFU analysis. Three animals and culture replicates per condition, *n* = 3. Error bars represent standard deviation. Nonparametric Kruskal–Wallis one-way analysis of variance with uncorrected Dunn’s multiple comparison test was performed. Indicated *P* values, *, *P*, 0.05; **, *P*, 0.01; ***, *P*, 0.005; ****. (C) SEM of *C. albicans* biofilms grown on coverslips in control and human EV inhibitor-treated biofilms (8  µg/mL) [omeprazole, simvastatin, or imipramine]. Arrows highlight the blue pseudocolored extracellular matrix. Scale bars: 40 µm. (D) Quantitative analysis of EV concentration in *C. albicans* biofilms in a 6-well biofilm assay using NTA following treatment with a human EV inhibitor (8 µg/mL) [simvastatin, omeprazole, or imipramine]. Each dot represents an independent biological replicate and reflects the mean of three technical replicates. Error bars denote standard deviation. A nonparametric Kruskal–Wallis one-way analysis of variance with an uncorrected Dunn’s multiple comparison test was performed, with a significant *P* value indicated *, *P*, 0.05; **, *P*, 0.01; ***, *P*, 0.005; ****. (E) The percent reduction in biofilm formation was measured using the XTT assay following treatment with fluconazole (1,000  µg/mL) plus a human EV inhibitor (8 µg/mL) [simvastatin or omeprazole] alone or combined with *C. albicans* biofilm EVs (4.3 × 10^6^ ± 1.4 × 10^5^ particles/mL) after 48 h of growth. Each dot represents an independent biological replicate and reflects the mean of three technical replicates. Error bars denote standard deviation. A nonparametric Kruskal–Wallis one-way analysis of variance with an uncorrected Dunn’s multiple comparison test was performed, with a significant *P* value indicated *, *P*, 0.05; **, *P*, 0.01; ***, *P*, 0.005; ****.

To analyze the impact of mammalian vesicle inhibitors on *Candida* biofilms, we examined each drug alone and in conjunction with fluconazole using a checkerboard format with an XTT assay as an estimate of remaining viable burden. Untreated *Candida* biofilms tolerated concentrations of fluconazole 1,000 µg/mL (approximately 1,000-fold greater than planktonic cells). Modest concentrations of each agent (8 µg/mL) combined with high concentrations of fluconazole (1,000 µg/mL) exerted reductions in viable burden compared to monotherapy, which minimally impacted biofilm burden (Fig. 1A). We observed a dose-response relationship for fluconazole + extracellular vesicle (EV) inhibitor combinations over an EV inhibitor concentration range of 0.5–256 µg/mL. EV inhibitor monotherapy treatments exhibited antifungal effects (>20% change in biofilm burden) at relatively high concentrations (64 µg/mL for omeprazole and 128 µg/mL for simvastatin) (Fig. S1A and B). This is consistent with previously observed antifungal effects for these agents at high concentrations (16–19).

To further elucidate the potential therapeutic value of these drug interactions, we utilized a rat central venous catheter model that mimics a clinically relevant biofilm infection (Fig. 1B). Based on the *in vitro* studies, we selected the most effective drug from each EV inhibitor group (exosome biogenesis-simvastatin and EV release-omeprazole). Following the establishment of mature biofilm, we instilled each drug alone and combined with fluconazole for 24 h. We then removed catheters and assessed treatment efficacy by measuring the remaining *Candida* viable catheter burden. For both EV inhibitors, the drug combination therapies reduced viable burden to a greater extent than monotherapy.

Extracellular matrix production, a canonical feature of biofilms, is necessary for community fortification and protection from antifungals. To examine how the mammalian EV inhibitors influence biofilm matrix, we used scanning electron microscopy to estimate biofilm matrix with an *in vitro* coverslip model (Fig. 1C). We selected three mammalian inhibitors (omeprazole, simvastatin, and imipramine) for study at 8 µg/mL and observed marked reductions in visible biofilm matrix (see arrows) following a 24 h exposure. This suggests that the enhanced efficacy associated with these therapies is due to a matrix disruption action.

We next assessed the impact of three mammalian inhibitors on EV production during fungal biofilm growth (Fig. 1D). Following exposure to the mammalian EV inhibitors at 8 µg/mL, we observed a two- to fourfold reduction in biofilm EV production. This effect was dose-dependent with nearly complete abrogation of biofilm EV production at the highest concentrations studied (Fig. S2A through C). As biofilm EVs deposit the extracellular matrix, this finding is consistent with the visual loss of matrix (Fig. 1C).

Previous investigations have found that the addition of exogenous extracellular vesicles to matrix-depleted *Candida* biofilms can restore matrix function (7). To test the theory that the activity of the omeprazole- and simvastatin-fluconazole combination therapy is EV-dependent, we performed vesicle add-back experiments (Fig. 1E). Remarkably, the addition of EVs from untreated biofilms to the treated biofilms restored a significant degree of drug resistance to the biofilm community. The finding that drug resistance was not fully restored raises the possibility of other non-EV-dependent pharmaceutical effects. It is also possible that the endogenous EVs did not fully saturate the system during the administration time. However, the sum of the findings shows that mammalian EV inhibitors impact fungal biofilm matrix production and drug resistance primarily through an EV-dependent mechanism. Our observations provide proof of principle that EV-based therapeutics may be a useful platform for anti-biofilm strategies, particularly if they could be targeted to biofilms or selectively designed against fungal machinery. While the concentrations of these FDA-approved inhibitors utilized in these studies are near the maximal systemic concentration, catheter lock therapy with the drugs would be a clinical possibility combined with the antifungal fluconazole.

## SUPPLEMENTAL MATERIAL

Supplemental material aac.00045-25-s0001.pdf Figures S1 and S2 and supplemental methods.

ASM does not own the copyrights to Supplemental Material that may be linked to, or accessed through, an article. The authors have granted ASM a non-exclusive, world-wide license to publish the Supplemental Material files. Please contact the corresponding author directly for reuse.
