# Supplementary material for: Vesicle inhibition reduces Candida biofilm resistance
Source: Antimicrob Agents Chemother. 2025 Mar 26;69(5):e00045-25. doi: 10.1128/aac.00045-25 (PMC12057333; doi:10.1128/aac.00045-25)
Supplement: Supplemental material — Figures S1 and S2 and supplemental methods. [file aac.00045-25-s0001.pdf]

Supplemental Information

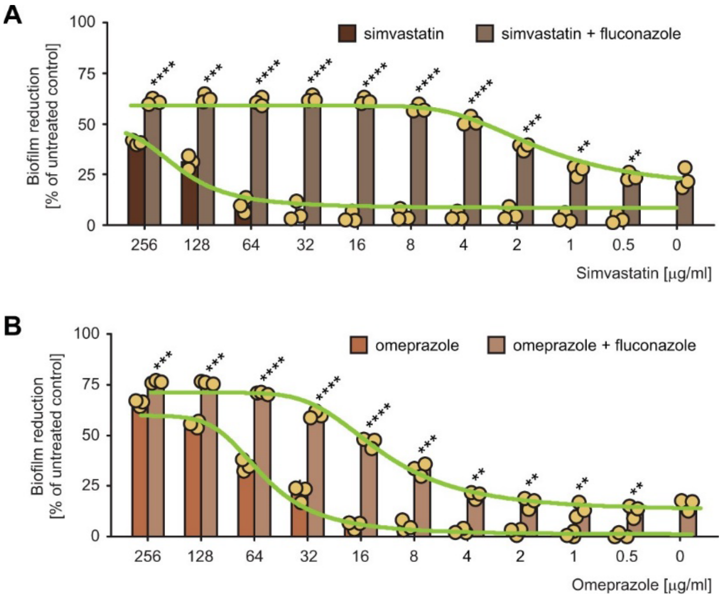

Figure 1S. **Impact of EV inhibitors on fluconazole *C. albicans* biofilm activity in vitro** (**A**) The percent reduction in biofilm formation was measured using the XTT assay following treatment with either fluconazole (1000 µg/ml) or a human EV inhibitor (0.5-256 µg/ml). **A.** simvastatin **B.** omeprazole alone or combined, compared to untreated biofilms. Each dot represents an independent biological replicate and reflects the mean of 3 technical replicates. Error bars denote standard deviation. A non-parametric Kruskal–Wallis one-way analysis of variance with an uncorrected Dunn's multiple comparison test was performed. Indicated p values, *, P , 0.05; **, P , 0.01; ***, P , 0.005; ****.

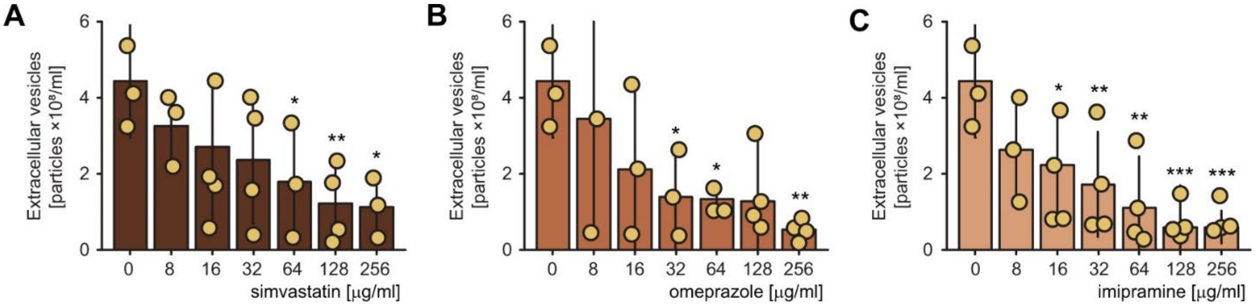

Figure 2S **Impact of EV inhibitors on *C. albicans* biofilm EV production in vitro** Quantitative analysis of EV concentration in *C. albicans* biofilms in a 6-well biofilm assay using NTA following treatment with a human EV inhibitor (8 µg/ml) [**A.** simvastatin, **B.** omeprazole, or **B.** imipramine]. Each dot represents an independent biological replicate and reflects the mean of 3 technical replicates. Error bars denote standard deviation. A non-parametric Kruskal–Wallis one-way analysis of variance with an uncorrected Dunn's multiple comparison test was performed, with a significant *p*-value indicated *, P , 0.05; **, P , 0.01; ***, P , 0.005; ****.

METHODS

Strains and media. *C. albicans* SN250 was utilized for all studies. The strain was sustained on yeast extract-peptone-dextrose (YPD) medium with uridine. Biofilms were grown in RPMI 1640 buffered with 4-morpholinepropanesulfonic acid (MOPS).

In vitro biofilm models. Three in vitro biofilm models were used, including a 96-well and 6-well polystyrene plate, or glass coverslip[1]. Biofilm drug susceptibility and vesicle production were assessed using the 96-well polystyrene plate assay using an XTT assay[1]. Matrix assessment utilized SEM imaging of coverslip grown biofilms after 24h of growth.

Extracellular vesicle isolation. EVs were isolated from biofilms grown in 6-well polystyrene plates. Media was removed from the plates, filter sterilized, and concentrated using a Vivaflow 200 unit (Sartorius AG, Goettingen, Germany) equipped with a Hydrosart 30 kDa cut-off membrane. The sample was centrifuged at 10,000 × *g* for 1 h at 4°C to remove cellular debris. The pellets were discarded, and the supernatant was centrifuged again as described above. This supernatant was then centrifuged at 100,000 × *g* for 1.5 h at 4°C. The supernatants were discarded, and the pellet was resuspended in phosphate-buffered saline (PBS) (pH 7.2) before being re-centrifuged at 100,000×*g* for 1 h at 4°C. The extracellular vesicles collected were further purified using flash size-exclusion chromatography on a qEV/35 nm column (Izon Science). Finally, the vesicles were filter-sterilized and stored at 4°C for future use.

Quantitative extracellular vesicle analysis. Exosomes were quantified using nanoparticle tracking analysis (NTA)[2]. EV samples were diluted in PBS to a final volume of 1 ml and pretested to obtain an ideal 30-100 particles per frame rate using a NanoSight NS300 system coupled with an autosampler (Malvern). The following settings were applied: camera level was increased to 16 and camera gain to 2 until tested images were optimized and nanoparticles were distinctly visible without exceeding particle signal saturation. Each measurement consisted of five 1-min videos with a delay of 5 s between sample introduction and the start of the first measurement. For detection threshold analysis the counts were limited to 10-100 red crosses and no more than 5-7 blue crosses. Acquired data were analyzed using the NanoSight Software NTA 3.4 Build 3.4.003. At least 1000 events in total were tracked per sample in order to minimize data skewing based on single large particles.

In vitro biofilm SEM imaging. In vitro biofilms were grown on coverslips in 6-well plates. Ten microliters of fetal calf serum were placed on each coverslip and dried for 1 h. Forty microliters of an inoculum of 10^8 cells/ml was placed on the coverslip and incubated at 37°C for 24 h. Following incubation, the cells were fixed with 4% formaldehyde and 1% glutaraldehyde at 4°C overnight. Coverslips were then washed with PBS and treated with 1% osmium tetroxide for 30 min at 22°C. Samples were subsequently washed with a

series of increasing ethanol dilutions (30 to 100%), followed by critical point drying and coating with platinum. SEM of samples was performed using a LEO 1530 microscope.

In vivo biofilms were propagated in a rat (female, 400 g Sprague-Dawley) central venous catheter biofilm model as previously described[3]. After a 48-h biofilm formation phase, the devices were removed, sectioned to expose the intraluminal surface, and processed for SEM imaging as described above.

In vitro biofilm and planktonic antifungal susceptibility assay. In vitro biofilm drug susceptibility to was assessed using a tetrazolium salt XTT reduction assay. The percent reduction in biofilm growth compared to untreated controls is reported. The CLSI M27 A3 and broth microdilution susceptibility methods were used to determine activity against planktonic *Candida*.

In vivo rat central venous catheter biofilm model. A jugular vein rat catheter infection model was utilized for in vivo biofilm assessments (Envigo, Indianapolis, IN)[3]. Quantitative cultures of *C. albicans* after 24 h of in vivo growth were utilized to measure viable cell burden. For drug treatment, fluconazole (250 μg/ml), simvastatin or omeprazole (32 μg/ml) alone or in combination were instilled and dwelled in the catheter over a 24-h period. The post treatment viable burden was compared to untreated controls.

EV add-back assay. Biofilms were propagated in the wells of 96-well plates. After a 6-h biofilm formation period, the biofilms were washed with PBS twice. Biofilms were then treated with fluconazole (1000 μg/ml), or the combination of fluconazole and either omeprazole or simvastatin (64 μg/ml). Exogenous purified EVs collected from 48h biofilms were administered at a concentration of $4.3\times10^6\pm1.4\times10^5$ particles/ml 1 hour before antifungal therapy for one series of wells[4]. Following another 24h of incubation, the quantity of *Candida* biofilm cells was assessed using the XTT assay. The results are presented as a percent reduction by comparing untreated biofilms with those that were treated.

Statistical Analysis. Data sets were analyzed using the one-way analysis of variance (ANOVA) and the post-hoc Tukey HSD test.

Ethics Statement. Animal procedures were approved by the Institutional Animal Care and Use Committee at the University of Wisconsin (protocol DA0031).

References

1 Ramage, G. & Lopez-Ribot, J. L. Techniques for antifungal susceptibility testing of Candida albicans biofilms. *Methods Mol Med* **118**, 71-79, doi:10.1385/1-59259-943-5:071 (2005).
2 Gardiner, C., Ferreira, Y. J., Dragovic, R. A., Redman, C. W. & Sargent, I. L. Extracellular vesicle sizing and enumeration by nanoparticle tracking analysis. *J Extracell Vesicles* **2**, doi:10.3402/jev.v2i0.19671 (2013).
3 Andes, D. *et al.* Development and characterization of an in vivo central venous catheter *Candida albicans* biofilm model. *Infect Immun* **72**, 6023-6031 (2004).
4 Zarnowski, R. *et al.* Candida albicans biofilm-induced vesicles confer drug resistance through matrix biogenesis. *PLoS Biol* **16**, e2006872, doi:10.1371/journal.pbio.2006872 (2018).
